# Physical Activity and Stress of Children and Adolescents during the COVID-19 Pandemic in Germany—A Cross-Sectional Study in Rural Areas

**DOI:** 10.3390/ijerph19148274

**Published:** 2022-07-06

**Authors:** Michael Braksiek, Uta Lindemann, Iris Pahmeier

**Affiliations:** 1 Department of Sport Science, University of Vechta, 49377 Vechta, Germany; michael.braksiek@uni-vechta.de (M.B.); iris.pahmeier@uni-vechta.de (I.P.); 2 Department of Sport Science, Bielefeld University, 33615 Bielefeld, Germany

**Keywords:** COVID-19, physical activity, mental health, stress and well-being, children and adolescents

## Abstract

Although infection with SARS-CoV-2 appears to be less dangerous for children and adolescents, research indicates that the measures to contain the COVID-19 pandemic have had and continue to have negative effects on children’s and adolescents’ mental health and physical activity (PA). Due to the different health policies, country-specific studies as well as studies in different phases of the pandemic are important to obtain a differentiated picture of the effects of the pandemic. This study set out to investigate children’s and adolescents’ PA, stress, and well-being as well as the associations between these two variables during a phase of a gradual decline in measure to contain the pandemic in Germany. For this purpose, 1293 children and adolescents in a rural area of a federal state in Germany were investigated. The results indicated that children and adolescents felt sadder and less well during this period than children and adolescents before the pandemic. Results also revealed that they were more physically active than children and adolescents before the pandemic as well as in the second lockdown but less active than in the first lockdown. Both well-being and sadness were positively associated with the PA. The study contributes to a more comprehensive understanding of the situation of children and adolescents during the COVID-19 pandemic.

## 1. Introduction

The outbreak of the COVID-19 pandemic led to an unprecedented health crisis that resulted in many deaths [1]. A variety of measures were taken to contain the pandemic. Some of these measures were temporary but very drastic (e.g., lockdowns, quarantine), while others were designed to last for a longer period and, in some cases, have become part of public life (e.g., masks, testing). The measures to contain the virus affected all population groups—elders, adults, adolescents, and children. Although infection with the virus appears to be less dangerous for children and adolescents, research indicates that the measures to contain the virus have had and continue to have negative effects on children’s mental health and physical activity [2,3,4,5].

A steadily growing number of studies have demonstrated negative effects on various aspects of children’s and adolescents’ mental health. In particular, the prevalence of posttraumatic stress symptoms, sleep disorders, anxiety, and depression symptoms increased [6]. This increase is about an average doubling of these symptoms [7]. Specifically related to the pandemic, children were found to have fear and concerns about the impact of the pandemic on their lives [3]. In addition to cross-sectional studies, some longitudinal studies were able to investigate the development of mental health in children and adolescents over the course of the pandemic [8,9]. In Germany, the representative COPSY-study (COVID-19 and Psychological Health) analyzed the health-related quality of life (HRQoL) and mental health of children and adolescents during the pandemic at three measurement points [7,10]. The first wave of the study was conducted during a partial lockdown (May–June 2020), the second wave during a nationwide lockdown (December 2020–January 2021), and the third wave during a period with very few restrictions (September–November 2021). Compared to pre-pandemic data, the HRQoL of the children and adolescents decreased significantly in wave 1 and wave 2. In wave 3, the HRQoL increased slightly but remained at a low level [7]. A similar picture emerged regarding mental health. During the first two waves, mental health problems, anxiety, and depression symptoms increased and decreased slightly in wave 3 [7]. In accordance with these results, Vogel et al. [11] showed that physical and mental well-being in German children and adolescents was significantly lower during the first lockdown (April 2020) than before the pandemic. These results are mostly in line with other longitudinal studies investigating children and adolescents in other countries [12]. Besides these global (e.g., HRQoL) and psychopathology (e.g., depression, conduct problems) aspects of mental health, few studies focused on psychological stress and certain stress symptoms as early indicators of pathopsychological mental health problems [13,14]. For example, Achterberg et al. [8] showed that children who were more stressed in lockdown showed a stronger increase in externalizing behavior (e.g., hyperactivity), demonstrating the relationship between stress and mental health during the pandemic. Paschke et al. [15] showed in a representative sample that German adolescents perceived significantly more stress during the first lockdown than before. More differentiated analyses also indicated that children and adolescents in urban areas had poorer mental health status than children and adolescents in rural areas [16,17]. Although the body of research on children’s and adolescents’ mental health during the pandemic is growing, there are still few studies on the stress experienced by children and adolescents during the pandemic. However, studies on this topic are important to analyze stress as an early indicator of mental health problem that could arise in the future. 

Besides psychological health aspects, the measures to contain the pandemic affect behavioral health aspects as well. Especially, the closure of schools and sports facilities and the call for social distancing had a negative effect on the physical activity (PA) level of children and adolescents, reenforcing the pandemic of global physical inactivity [5,18]. Physical inactivity is associated with several negative health outcomes such as, for example, obesity and pre-adiposity as well as poor cardiovascular fitness and blood levels [19,20]. A recent review and meta-analysis of longitudinal studies investigating changes in PA during the pandemic revealed mixed results for the PA of children and adolescents, resulting in a negative but non-significant effect of the pandemic on the PA levels of children and adolescents [21]. Nevertheless, including studies with mixed results, most studies in meta-analysis reported a decrease in PA in children and adolescents. Additionally, cross-sectional studies investigating this topic mostly revealed a decrease of children’s and adolescents PE levels [5,22]. Most likely, the ambiguous results arise from different measures in different countries to contain the pandemic. A specific and unique effect was revealed in the analysis of PA of children and adolescents in Germany during the first lockdown (April 2020). There, the representative German Motoric-Modul (MoMo) cohort study examined the PA of children and adolescents even before and during the pandemic [23]. On one hand, the study showed an increase in total PA, especially in non-organized sports [24]. On the other hand, a decrease in organized PA (e.g., sport clubs) was shown at the same time. This effect was presumably due to the additional recreational time owing to school closures and the closure of sport clubs during the lockdown. In the second lockdown, however, the high PA levels could not be maintained and partly fell below the pre-pandemic level [23]. Furthermore, differentiated analyses revealed that the effect of the increase in PA in the first lockdown depended on population density [25]. The higher the population density where the children and adolescents lived, the lower the increase in PA. This effect was also shown in studies from other countries [22]. Summarizing, studies tend to indicate a decrease in PA of children and adolescents during the pandemic, but context factors (e.g., population density, type of restrictions) moderate this effect. 

Since the relationship between PA and mental health in children and adolescents is well-established [26,27], studies have also examined this relationship in the pandemic, as the interactions between these two factors may be protective against influences of the pandemic situation [28,29]. Despite the fact that the causality of the relationship is not as clear-cut as often assumed [30,31,32], most studies postulate a positive effect of PA on mental health. Cross-sectional studies also basically demonstrated the relationship between PA and mental health of children and adolescents in the pandemic as well [28,33]. However, there are hardly any longitudinal studies that examined the causality of this relationship during the pandemic. Based on data from Momo, Wunsch et al. [34] showed that the HRQoL of children and adolescents before the pandemic had a positive effect on the PA during the first lockdown, whereas the PA before the pandemic had no effect on the HRQoL during the first lockdown [34]. This was especially evident in girls and young children (4–10 y). Hence, the relationship between mental health and PA is evident also in the pandemic, but the causality of this relationship in this context is not clear. 

Generally, it can be stated that children’s and adolescents’ mental health and PA as well as their relationship has been scientifically evaluated during the pandemic. Nevertheless, the number of studies is scarce, especially compared to studies in adults and elders [3,33,34]. In particular, children under the age of ten were rarely investigated [3,34]. Regarding mental health, the studies are partly of low quality because non-validated instruments were used [3]. Additionally, focus was mostly on global (e.g., HRQoL) and psychopathology (e.g., depression) aspects of mental health. Overall, studies on the mentioned topics mostly focused on phases of the pandemic with restrictive measures (e.g., lockdowns). Less is known about phases with less restrictive measures or phases in transition from restrictive to less restrictive measures. 

The present study took up these desiderata. The study focused on a phase after restrictive measures (i.e., lockdown) in Germany. The aim of the study was to (1) investigate children’s and adolescents’ stress symptoms as early indicators of mental health problems as well as (2) their PA as an indicator of physical health. Furthermore (3), the associations of stress and PA during this period was examined. Due to the very different health policies regarding the pandemic as well as different social structures, country-specific studies as well as studies in different phases of the pandemic are important to obtain a comprehensive and differentiated picture of the effects of the pandemic on children’s health. Thus, the study contributes to a better understanding of the effects of the measures containing the pandemic.

## 2. Materials and Methods

### 2.1. Data Collection 

Data were collected between the end of May and the middle of June 2021. The situation regarding measures to contain the pandemic was diffuse and dynamic [35]. There were regular changes in the restrictions, which were implemented at different speeds and in different ways. From the second lockdown in Germany (from the end of 2020), measures were gradually relaxed, and this also affected the sports sector. Until the beginning of March, offers of recreational and amateur sports were closed at public and private sports facilities. From then on, children under 14 were allowed to participate in outdoor sports in fixed groups of 10 children. From mid-May, this rule was extended to children up to 18 years of age and a fixed group of 30 children and adolescents. Indoor sports with more than two people from another household were only possible again from the beginning of May under strict hygiene regulations. The situation in schools was also diffuse and dynamic [36]. Physical education classes at schools were scarce and irregular. Children were still required to wear masks and keep a distance at the time of data collection. In some schools, classes were divided and taught weekly. 

The data of this study were gathered using online questionnaires and paper-pencil questionnaires. The questionnaires were completed during regular classes or distance learning. Children and adolescents in the third to tenth grades were surveyed. For the younger participants (primary school), only paper-pencil questionnaires were used so that they could answer the questions under supervision. Online questionnaires were used in some cases with older participants. The online survey was programmed on the platform www.soscisurvey.de. In both cases, the questionnaire was explained by the survey team based on a standardized manual. The team was always available to answer questions. Participation in the survey was voluntary, and informed parental consent was obtained prior to the survey. The surveyed classes were selected according to whether there was already contact between the respective school and the research team (e.g., by supervising students in internships). Therefore, the resulting sample is a convenience sample. This form of sampling is due to the pandemic situation and the resulting ad hoc nature of research in the pandemic, which also led to many other studies using convenience samples [5]. The final sample consisted of *N* = 1293 respondents (*M*_age_ = 11.0 years; *n*_female_ = 635) that were included in the empirical analysis.

### 2.2. Measures 

All data were collected anonymously. Distinct conclusions from the data to an individual participant were not possible.

#### 2.2.1. Demographics 

Gender and age of the participants were assessed via the questionnaire. The school grades, school types, and whether or not physical education lessons took place at the time were added to the data by the survey team. 

#### 2.2.2. Physical Activity 

To measure the physical activity of the participants, two items from the validated MoMo physical activity questionnaire were used [37]. First, participants were given an explanation of what is meant by physical activity (for detail, see Schmidt et al. [38]). After that, the first item asked for the number of days in which participants were physically active for at least 60 min during a typical week during COVID-19 (“Over a typical or usual week during the coronavirus pandemic, on how many days are you physically active for a total of at least 60 min per day?”). The second item asked for the number of days in which participants were physically active for at least 60 min during the last week (“Over the last week, on how many days were you physically active for a total of at least 60 min per day?”). This item was used to determine the adherence the WHO guidelines [39]. The items were chosen to ensure comparability with data from the representative MoMo data pre- and during COVID-19 [24,34].

#### 2.2.3. Stress and Well-Being

Stress and well-being were measured using four subscales from the Stress-Symptom and Well-Being Scales from the Stress and Coping Questionnaire for Children and Adolescents [40,41]. Three scales were used to capture stress symptomatology. The measured symptoms were anger, anxiety, and sadness. The fourth scale measured well-being. The scales assess stress symptomatology and well-being experienced in the last week. The participants had to indicate on a three-point scale (never, once, several times) how often they had certain emotional states in the last week (e.g., “How many times were you lonely in the last week?”). Each scale consisted of four items. The scales are validated for children and adolescents in third to eighth grade [41] and showed associations with psychopathological mental health indicators [40]. Scale reliability was assessed using Cronbach’s α, which was 0.83 for anger, 0.64 for anxiety, 0.74 for sadness, and 0.76 for well-being, indicating good to acceptable internal consistency [42].

### 2.3. Data Analysis 

All analyses were conducted using IBM SPSS 27 [43] and MPlus 7 [44]. Both items measuring physical activity were processed according to the MoMo protocol [38]: To obtain a value for the average physical activity (PA) of the participants, the mean values of both items was calculated. The values can be interpreted as average active days. To determine the WHO-guideline adherence, a dummy variable was calculated from the item capturing PA in the last week. Values between 0–6 were coded 0, and values above were coded 1. The stress and well-being scales were also processed according to the scale manual [45]. First, missing values in the stress and well-being scales were estimated when one response per scale was missing. Otherwise, the case would have been removed, but that was never necessary. The missing values were estimated using the EM-algorithm [46]. After that, a sum-score per scale was calculated. The analysis was completed in three steps. First, we descriptively compared the WHO guideline adherence with representative data from MoMo wave 3, lockdown study 1, and lockdown study 2. To ensure comparability, the sample was divided into gender and age groups (Table 1). In a second step, we investigated difference between the stress and well-being scores in the analyzed sample and representative norm data. To establish comparability with the norm data, the sample was divided into grade levels for this analysis. We used one-sample *t*-tests in SPSS 27 to investigate potential differences. To quantify the differences, Cohen’s d was calculated and common rules of thumbs were used for interpretation [47]. In a third step, we analyzed the associations between PA and stress and well-being calculating a regression model in MPlus 7. PA was set as the dependent variable, while stress and well-being were set as independent variables. Gender (dummy), physical education (dummy), and age were added as control variables. To consider the nested data structure (school classes), corrected standard errors that are robust to non-independence of the data were used in the analysis [44]. All variables in the regression model were checked for potential multicollinearity. All variation inflation factors were below 5, and tolerance values were above 0.2, indicating that multicollinearity was not an issue in the analysis [42]. Significance was set to *p* < 0.05 for all analyses. 

## 3. Results

### 3.1. Physical Activity 

The sample divided in age groups is presented in Table 1. The classification corresponds to the classification in MoMo [23]. The sample was evenly distributed in terms of gender. Regarding age, elementary school children are overrepresented.

Table 2 shows the average active days of the participants and the percentage of those who meet the WHO guidelines of being active for at least 60 min every day in the week. Descriptively, active days do not differ much between genders. Both the number of active days and the guideline adherence decreased with increasing age.

Table 3 relates the PA data to comparative data from MoMo [23]. In the youngest age group (6–10 y), the proportion of those who meet the guidelines was between the peak in lockdown 1 and the lower proportions in MoMo wave 3 and lockdown 2 for both genders. The same applies to the boys in the middle age group (11–13 y). The proportion of girls in this group who meet the guidelines was slightly higher than in lockdown 1. The average proportion fell between lockdown 1 and Momo wave 3 and lockdown 2. The same can be seen in the oldest age group (14–17 y) in relation to the genders. Here, however, the average proportion is also higher than that of lockdown 1.

### 3.2. Stress and Well-Being

Since the norm values for the comparison with the present data refer to grade levels, the sample was divided differently compared to the analysis of PA. The division into class levels is shown in Table 4. In a few primary school classes, teachers asked for a shorter questionnaire. As a result, the scale for anger and anxiety were removed. Thus, the scale for sadness could be left in the questionnaire to measure at least one stress symptom. The sample size in the grades 3 and 4 was therefore reduced from 838 to 645 regarding the analysis of anger and anxiety.

Table 4 also shows the scores of the stress-symptom and well-being scales as well as their comparison with representative norm values [45]. Since there were no norm values for the ninth and tenth grades, no comparisons could be made for this group. The comparisons of the values for anger and anxiety showed few significant differences with small effect sizes. Boys in the third and fourth grade had slightly higher values for anger. Girls in the third and fourth grade had somewhat lower values for anxiety but higher values in seventh and eighth grade. The results for sadness and well-being were clearer. Apart from girls in third and fourth grade, all groups showed significantly higher scores for sadness, which means that most of the children were sadder than the pre-pandemic average at the time of data collection. The effect sizes were in the small-to-medium range. Regarding well-being, all groups showed significantly lower scores with medium-to-large effect sizes, meaning children felt much less comfortable than the pre-pandemic average.

### 3.3. Associations of Physical Activity and Stress and Well-Being

Turning to the third research question, Table 5 shows the results of the regression analysis with PA (active days) as dependent variable. Due to the smaller sample size and the insignificant findings in comparison with the norm values, anger and anxiety were not included in the analysis. As an additional test for multicollinearity, the correlation of sadness and well-being was calculated. A negligible correlation was found (*r* = −0.28, *p* < 0.05) so that multicollinearity could still be precluded. Regarding the control variables, only age was negatively associated with PA. This result was already indicated in the descriptive statistics (Table 2). Gender and PE were not related with PA. Controlling for gender, age, and PE, both sadness and well-being were positively associated with PA. The standardized effects of sadness and well-being were about the same strength. The whole regression model explained a significant part (3.4%) of the children’s and adolescents’ PA. 

## 4. Discussion

This research set out to investigate children’s and adolescents’ PA, stress, and well-being as well as the associations between these two variables during a phase of a gradual decline in measure to contain the COVID-19 pandemic. Studies on these aspects at this stage in the pandemic are scarce to date. Likewise, there are few data on the age group studied here, particularly children under the age of 10.

Regarding the comparison of the PA data with data from Momo, the proportion of children and adolescents who adhered the WHO guideline of at least 60 min moderate PA per day during the phase of gradual decline of the measures to contain the pandemic were higher than the proportion before the pandemic in all age groups. This fact could have similar reasons as the considerably increased PA in the first lockdown in Germany [24]. Due to the still present restrictions in organized sports and the partial cancellation of classes, the children adolescents had more recreational time to do sports than before the pandemic. Since the data were collected at about the same time of year as the first lockdown, the weather conditions and thus the opportunities for outdoor sports are comparable. Even though the measures were less restrictive and slowly decreasing, the PA was lower than in lockdown 1 but also higher than in lockdown 2. The low level of PA in the second lockdown may be related to the limited opportunities for outdoor sports due to the poor weather conditions in January and February [23]. The higher PA seen in the present data can be attributed to both rising temperatures and a decrease in restrictive measures. For example, organized sports activities were possible again, at least those taking place outdoors. However, the fact that PA did not reach the level of the first lockdown could be because of the more structured days (e.g., less distance learning, more time in school), thus resulting in less recreational time. Recall that population density had an effect on the increase of PA during the first lockdown [25]; this could also be a reason for the high PA in this study. The data were collected in rural areas with a low population density, resulting in more public places and facilities for outdoor sport activities that could be used for organized as well as nonorganized PA. Comparable data from other countries at a time with a similar gradual decline of measure are seemingly not available. 

Concerning the children’s and adolescents’ stress and well-being, the comparison with the norm values gathered before the pandemic revealed that results were different regarding stress symptoms but consistent across age groups. Generally, the children and adolescents did not report more anger or higher anxiety compared to the norm values. Following common definitions, anger is linked with the urge to injure some target [48]. As the coronavirus, the pandemic, or the restrictive measures are highly abstract phenomena for children and adolescents, they might not have a concrete target against which to direct their potential anger. Moreover, striving to injure a target also implies some notion of how to injure the target. Given the abstractness of potential targets (e.g., the virus), children and adolescents may also lack this notion. Both considerations could be reasons for the average anger of the children and adolescents. The average anxiety values seem contradictory at first. While there are no comparative data from before the pandemic, studies report that children are afraid of COVID-19 [49,50]. Data from the COPSY-study revealed an increase in anxiety symptoms in the first and second lockdown in Germany but no increase at the third measurement point [7]. However, the average anxiety values in the present study can be explained by the fact that the data were collected after the second lockdown and especially after the start of the vaccination campaign [51]. There is evidence that children and adolescent are more afraid of infecting persons close to them (e.g., parents or grandparents) than of infecting themselves with the virus [11,52,53]. The present results indicated that these fears have been mitigated by the onset of population immunization through vaccination, which is a new insight regarding children’s and adolescents’ mental health during the pandemic. 

Regarding the children’s and adolescents’ sadness and well-being, results are clear and in line with other studies. The well-being of the children and adolescents was significantly lower than the average pre-pandemic well-being. This result echoed the findings of other studies conducted in phases with restrictive restriction [11,12] and showed that low well-being persisted even during periods of decline in the measures. Combined with data from the COPSY-study that showed an increase in HRQoL from the second lockdown (December/January) through September/October [7], the results suggest that it will take time to implement a reduction in measures and that it will take time to influence the well-being of children and adolescents. The results on sadness showed that even in a phase of declining restrictions, children and adolescents are still sadder than before the pandemic. These results extend findings from other studies. Other studies have demonstrated an increase in depressive symptoms during periods of more restrictive measures [7,50,54]. The reason for both findings may be the limited social contacts, lack of leisure activities, and the perception of stress in the immediate environment (e.g., situation of parents) [8,55]. The present data imply that less psychopathological aspects (i.e., sadness) are also affected by the pandemic. This is particularly relevant because sadness as a symptom of stress may be an indicator of serious mental disorders [56]. 

In the regression model for analyzing the associations between PA and stress and well-being, the control variables had different effects. Whereas the negative effect of age was in line with other studies during [21] and before the pandemic [57,58], the insignificant effect of gender needs to be considered more in detail. Most studies showed that boys are more physically active than girls, but this difference increases with age [57,58]. Representative data from Germany revealed that the gender differences regarding the adherence of the WHO guideline for PA do not become apparent until the age of 14 [57]. Accordingly, the lack of a gender effect in the present study can be explained by the fact that children aged 6–10 are overrepresented in the sample. Whether children and adolescents had physical education (PE) classes at school at the time of data collection did not affect their PA. This could be because the time for PA in PE classes is mostly less than 60 min as well as because the PE classes included less PA due to the existing obligations to wear masks and keep a distance. Regarding the stress symptoms, the positive association of well-being with PA is in line with other studies investigating the relation of mental health and PA during the first year of the pandemic [33]. Recall that, as the causality of this relationship is not clear, it can be interpreted in both ways. However, following results from Wunsch et al. [34], who showed a positive effect from pre-COVID-19 HRQoL on peri-COVID-19 PA and no effect in the different direction, higher well-being of children and adolescents in the present study might also have led them to be more physically active. Although causality cannot be established in this study, the positive association of the two variables (i.e., PA and well-being) is positive from a health perspective, as both variables are beneficial for the physical and mental health of the children and adolescents. 

The analysis also revealed a positive association between the children’s and adolescents’ sadness and their PA. Since sadness and well-being are negligibly correlated, both effects can be discussed separately. The sadness of children and adolescents has been hardly investigated during the pandemic. From a psychopathological perspective, the focus was primarily on depression. Most studies showed a negative correlation between depressive symptoms and PA in children and adolescents during the first year of the pandemic [28,33]. Pre-pandemic data revealed mixed results. For example, J.V. Ahn et al. (2018) as well as C.R. Nigg et al. (2021) measured children’s and adolescents’ emotional symptoms with a scale from the Strength and Difficult Questionnaire [59]. This dimension (i.e., emotional symptoms) has some similarities with the construct of sadness. Ahn et al. [60] could not show an effect of PA on children’s emotional symptoms, whereas Nigg et al. [31] showed a negative effect of children’s and adolescents’ emotional symptoms on their PA over a period of several years. The positive association of sadness and PA in the present study could be an indication of children and adolescents using PA as a coping strategy [61,62]. In contrast to the studies that showed a negative association of depression and PA [33], the data in this study were collected at a time during the pandemic when more PA was possible due to a decline of restrictive measures. The results suggest that the sadder the children and adolescents were, the more they seem to use the regained opportunities for sports activities. 

This study examined the PA and stress and well-being of children and adolescents in a rural area in Germany during a period of a gradual decline in measure to contain the COVID-19 pandemic. Children and adolescents are among a vulnerable group that suffers from pandemic-related restrictions. It is important to analyze the effects of measures to contain the pandemic on the mental and physical health of this population group to be able to make evidence-based decisions for the benefit of the health of this population group in comparable situations (e.g., new waves of infection due to mutations of the SARS-CoV-2-virus or pandemics by other viruses). Hence, understanding how children and adolescents react in different periods of the pandemic to different measures is important for health policy makers, educators, and parents (e.g., regarding the organization of psychological support for children and adolescents or the amount of time for PA in schools). Moreover, the analysis of the associations between mental health (i.e., stress) and physical health indicators (i.e., physical activity) during a pandemic provides additional information for health policy makers concerning decision about closing and opening parts of the public sports system. 

Nevertheless, the study has some limitation that can guide further research. First, the sample of the study was a convenience sample. Therefore, only children and adolescents in rural areas were surveyed, and the sample consists to a large extent of children between the age of 6 and 10, which makes it not representative for Germany. Second, the stress and PA data are based on self-reports by the children and adolescents. Although validated scales were used, and the survey teams assisted during the data collections, objective measures of PA and an assessment of the parents on the stress symptoms of the children might provide more detailed and more reliable information. In addition, the restrictions and their implementations as well as environmental conditions (e.g., weather) were variable over the period of data collection so that not exactly the same conditions were applied to all children and adolescents surveyed. Third, as the study was cross-sectional, empirical analyses of causal relationships (i.e., stress and PA) as well as within-person analyses of the development of children’s and adolescents’ stress and PA over the course of the pandemic were not possible. 

## 5. Conclusions

The present study adds to the existing literature about children’s and adolescents’ mental health and PA during the COVID-19 pandemic. It is, to our knowledge, the first study that investigated these variables in a period of a gradual decline of measures after the restrictive measures took place. It also contributes to better knowledge and understanding of the health status of children under the age of 10, as this age group has rarely been studied during the pandemic. Furthermore, the results revealed that a decline of measures and other important measures (vaccination) seem to influence children’s and adolescents’ stress and well-being. Additionally, the study provides initial evidence that some children and adolescents use the regained opportunities for physical activity more intensively and use them as a coping strategy against stress as well. The findings on sadness and well-being emphasize the need to create opportunities for PA in the pandemic and, in the event of a new wave of infection or new pandemic, to ensure that these continue for as long as possible. Despite the fact that the study is cross-sectional and used an ad hoc sample, it adds to the existing literature about the mentioned topics especially due to the specific measurement point. Therefore, it can be used in comprehensive reviews and meta-analysis reconstructing the effects of different measures in the course of the pandemic children’s and adolescents’ mental health and PA. 

Finally, the study contributes to a more comprehensive understanding of the situation of children and adolescents during the COVID-19 pandemic. In conjunction with evidence from other studies, the results can form a basis for the evidence-based decisions of health and education policy makers regarding future challenges.

## Figures and Tables

**Table 1:** Sample divided by age and gender.

Characteristics	Age Groups			
	6–10 Years	11–13 Years	14–17 Years	6–17 Years
Gender				
Male	418	93	147	658
Female	391	96	148	635
Total	809	189	295	1293
Age (*M*, *SD*)	9.4 (0.7)	11.5 (0.7)	15.3 (0.9)	11.0 (2.6)

Note: *M*, mean; *SD*, standard deviation.

**Table 2:** Active days and WHO guideline adherence divided by age and gender.

Activity	6–10 Years			11–13 Years			14–17 Years		
	Boys	Girls	Ø	Boys	Girls	Ø	Boys	Girls	Ø
Active Days *M* (*SD*)	4.3 (2.2)	4.1 (2.2)	4.2 (2.2)	4.0 (1.8)	3.9 (1.9)	3.9 (1.9)	3.8 (1.8)	3.8 (1.9)	3.8 (1.8)
WHO Guideline Adherence	32.3%	27.6%	30.0%	18.3%	20.8%	19.6%	15.0%	12.8%	13.9%

Note: *M*, mean; *SD*, standard deviation.

**Table 3:** Comparison of WHO guideline adherence in MoMo wave 3, lockdown 1, lockdown 2 [23], and phase of gradual decline of measures as a function of age and gender.

Characteristics	WHO Guideline Adherence				
Age Group	Gender	MoMo Wave 3 (2018–2020) (*N* = 1906)	Lockdown 1 (April–May 2020) (*N* = 1615)	Lockdown 2 (January–February 2021) (*N* = 1483)	Gradual Decline of Measures (End of May–Middle of June 2021) (*N* = 1293)
6–10	Boys	28.5%	44.6%	23.7%	32.3%
	Girls	21.8%	40.0%	23.8%	27.6%
	Total	25.3%	42.5%	23.8%	30.0%
11–13	Boys	13.8%	27.5%	13.3%	18.3%
	Girls	6.6%	17.9%	8.9%	20.8%
	Total	10.1%	22.6%	11.0%	19.6%
14–17	Boys	5.1%	17.0%	8.6%	15.0%
	Girls	6.5%	8.6%	6.3%	12.8%
	Total	5.8%	12.4%	7.4%	13.9%

**Table 4:** Comparisons of stress and well-being scores with norm values [45] as a function of gender and grades of school.

Stress And Well-Being	Gender	Grades			
		3rd–4th Grade (*M*_age_ = 9.6)	5th–6th Grade (*M*_age_ = 11.3)	7th–8th Grade (*M*_age_ = 14.6)	9th–10th Grade (*M*_age_ = 15.7)
Anger *M* (*SD*)	Boys	7.2 * (2.4)	7.8 (3.1)	7.8 (2.6)	8.5 (2.5) (*n* = 88)
		*d* = −0.14	*d* = 0.09	*d* = −0.07	
		(*n* = 331)	(*n* = 54)	(*n* = 64)	
	Girls	7.2 (2.3)	7.6 (2.7)	8.4 (2.7)	8.9 (2.5) (*n* = 91)
		*d* = 0.06	*d* = 0.052	*d* = 0.15	
		(*n* = 314)	(*n* = 58)	(*n* = 67)	
	Total	7.2 (2.3)	7.7 (2.9)	8.1 (2.6)	8.7 (2.5)
		(*n* = 645)	(*n* = 112)	(*n* = 131)	(*n* = 179)
Anxiety *M* (*SD*)	Boys	7.5 (2.6)	8.3 (2.8)	7.5 (2.3)	7.8 (2.0) (*n* = 88)
		*d* = 0.01	*d* = 0.10	*d* = −0.20	
		(*n* = 331)	(*n* = 54)	(*n* = 64)	
	Girls	7.7 * (2.1)	8.1 (2.6)	8.5 * (2.1)	7.8 (2.2) (*n* = 91)
		*d* = −0.15	*d* = 0.03	*d* = 0.25	
		(*n* = 314)	(*n* = 58)	(*n* = 67)	
	Total	7.6 (2.1)	8.2 (2.7)	8.0 (2.3)	7.8 (2.1)
		(*n* = 645)	(*n* = 112)	(*n* = 131)	(*n* = 179)
Sadness *M* (*SD*)	Boys	6.6 * (2.0)	6.4 (2.31)	7.0 * (2.4)	6.4 (2.3) (*n* = 88)
		*d* = 0.28	*d* = 0.18	*d* = 0.42	
		(*n* = 435)	(*n* = 54)	(*n* = 64)	
	Girls	7.2 (2.2)	7.9 * (2.5)	8.5 * (2.4)	8.9 (2.6) (*n* = 91)
		*d* = 0.09	*d* = 0.38	*d* = 0.43	
		(*n* = 403)	(*n* = 58)	(*n* = 67)	
	Total	6.9 (2.1)	7.2 (2.5)	7.8 (2.5)	7.5 (2.7)
		(*n* = 838)	(*n* = 112)	(*n* = 131)	(*n* = 179)
Well-Being *M* (*SD*)	Boys	11.1 * (1.5)	11.1 * (1.67)	10.4 * (1.86)	11.5 (1.1) (*n* = 88)
		*d* = −0.63	*d* = −0.57	*d* = −0.64	
		(*n* = 435)	(*n* = 54)	(*n* = 64)	
	Girls	11.2 * (1.34)	10.4 * (1.9)	9.9 * (2.2)	10.7 (1.7) (*n* = 91)
		*d* = −0.61	*d* = −0.86	*d* = −0.99	
		(*n* = 403)	(*n* = 58)	(*n* = 67)	
	Total	11.1 (1.4)	10.7 (1.8)	10.1 (2.0)	11.1 (1.5)
		(*n* = 838)	(*n* = 112)	(*n* = 131)	(*n* = 179)

Note: *M*, mean; *SD*, standard deviation; *d*, Cohen’s d; * *p* < 0.05.

**Table 5:** Regression results for physical activity.

Variables	*B*	*SE* (*B*)	β
Age	−0.091	0.037	−0.107 *
Gender ^a^	−0.208	0.161	−0.051
PE ^b^	−0.496	0.254	−0.107
Sadness	0.088	0.028	0.100 **
Well-Being	0.142	0.042	0.107 **
*R* ^2^	0.034 *		

Note: PE, physical education; ^a^ 0, male; 1, female; ^b^ 0, no PE; 1, PE. ** *p* < 0.01, * *p* < 0.05.

## Data Availability

The data are not publicly available due to local legislation.
